# Electroconvulsive therapy induces rapid microstructural and macrostructural changes, but microstructural changes are longer-lasting

**DOI:** 10.1007/s00406-026-02195-0

**Published:** 2026-03-02

**Authors:** Joey P. A. J. Verdijk, Julia C. M. Pottkämper, Freek ten Doesschate, Laurens A. van de Mortel, Sven Stuiver, Leif Oltedal, Paul J. Lucassen, Esmee Verwijk, Michel J. A. M. van Putten, Jeannette Hofmeijer, Guido van Wingen, Jeroen A. van Waarde

**Affiliations:** 1 Department of Psychiatry, Rijnstate Hospital , P.O. Box 9555, 6800 TA Arnhem, The Netherlands; 2 Department of Clinical Neurophysiology, University of Twente , Enschede, The Netherlands; 3 Department of Psychiatry, Amsterdam UMC location University of Amsterdam, Amsterdam, The Netherlands; 4 Amsterdam Neuroscience , Amsterdam, The Netherlands; 5 Department of Radiology, Mohn Medical Imaging and Visualization Center, Haukeland University Hospital , Bergen, Norway; 6 Department of Clinical Medicine, University of Bergen , Bergen, Norway; 7 Department of Neurology, Rijnstate Hospital , Arnhem, The Netherlands; 8 Brain Plasticity Group, Swammerdam Institute for Life Sciences, University of Amsterdam , Amsterdam, The Netherlands; 9 Department of Psychology, Brain & Cognition, University of Amsterdam , Amsterdam, The Netherlands; 10 Department of Medical Psychology, Neuropsychology, Amsterdam UMC, Amsterdam, The Netherlands; 11 ECT Department Haaglanden, Parnassia Psychiatric Institute , The Hague, The Netherlands

**Keywords:** Electroconvulsive therapy, Diffusion tensor imaging, Cognitive side-effects, Major depression, Neuroplasticity

## Abstract

**Background:**

Electroconvulsive therapy (ECT) is an effective treatment for major depressive episodes, though its mechanisms remain unclear. Its use is limited by stigma and potential cognitive side-effects. Repeated magnetic resonance imaging (MRI) may provide further insights into brain changes related to ECT’s efficacy and cognitive outcomes.

**Methods:**

We analyzed cognitive, efficacy, and MRI data from depressed patients undergoing ECT in two prospective longitudinal trials (NCT04028596, NL-OMON43040), using 26 matched healthy controls (2020-BC-12375) to control for test-retest effects. All participants were scanned on the same MRI system. Structural and diffusion tensor imaging (DTI) scans were acquired at baseline, after the initial ECT sessions, within two weeks post-treatment, and at three-month follow-up. Linear and nonlinear changes in grey matter (GM) volume, white matter (WM) volume, mean diffusivity (MD), and fractional anisotropy (FA) were assessed. Early brain changes were examined in relation to clinical outcomes.

**Results:**

After the 3rd ECT session (*n* = 19), subcortical GM volume increased with a right-lateralized MD decrease. Two weeks post-ECT (*n* = 30), cortical GM volume increased, returning to baseline by three months. In WM, MD increased linearly post-ECT and remained elevated at follow-up, with no widespread FA changes (*n* = 30). ECT transiently impaired verbal learning (*n* = 23, *p*_*bonferroni*_ = 0.04), letter fluency (*n* = 22, *p*_*bonferroni*_ = 0.04), and animal fluency (*n* = 62, *p*_*bonferroni*_ = 0.02). Early brain changes were not associated with cognitive or efficacy outcomes.

**Conclusions:**

These results suggest that ECT induces rapid changes in brain macrostructure and microstructure. While macrostructural increases are temporary, microstructural changes in brain structure are longer-lasting.

**Supplementary Information:**

The online version contains supplementary material available at 10.1007/s00406-026-02195-0.

## Introduction

Major depressive disorder (MDD) is a common mental disorder, with a lifetime prevalence of up to 20% [1]. Psycho- and pharmacotherapy benefit around 50% of patients, but many of them relapse and an estimated 30% of patients suffers from treatment-resistant depression (TRD) [2, 3]. A frequently used and generally effective treatment modality for TRD cases is electroconvulsive therapy (ECT) [4]. Yet, its use is limited by practicalities, variation in response rate, stigma, concerns over possible (cognitive) side-effects and a lack of mechanistic insight. As a result, less than 1% of depressed patients receives ECT [5–8].

Currently, the most common theories on the possible mechanisms of action of ECT describe an induction of immediate early genes, brain-derived neurotrophic factor, and neuroplastic effects (synaptogenesis, dendritic arborization, glial activation, angiogenesis and neurogenesis) [9–11]. These theories are mainly derived from animal models. Clarity about the mechanisms of action and safety may help reduce fear and promote a more effective utilization.

Macrostructural brain changes have been studied most extensively in ECT-patients. Using T1-weighted magnetic resonance imaging (T1W MRI), increased volumes of hippocampus and amygdala have been reported within 24 h after a first ECT-session [12, 13]. After a completed ECT-course generalized increases of grey matter (GM) volume are found [9]. These seem transient, as GM volume decreases again in three to six months after the ECT-course [14, 15]. How these macrostructural changes relate to the possible mechanisms of action of ECT is unclear, since neuroplastic effects are not expected to (fully) explain generalized increases of GM volume [9].

Microstructural brain changes have so far been more sparsely studied in ECT-patients. Diffusion tensor imaging (DTI) holds potential to further elucidate the nature of microstructural changes in both GM and white matter (WM). The few studies that applied DTI report opposing findings regarding WM changes of fractional anisotropy (FA) and mean diffusivity (MD) following completion of an entire ECT-course [16–18]. Current DTI evidence further indicates that MD decreases in hippocampal GM after the ECT-course, which has been hypothesized to be governed by neuroplastic changes [19, 20]. Until now, however, studies of diffusivity dynamics immediately following separate ECT sessions are lacking, which may provide additional insight into the nature of these microstructural changes.

In daily ECT-practice, patients often show rapid improvements in mood but also develop cognitive side-effects, that can last for weeks or longer [21]. Though inconsistent, earlier studies have shown indications that rapid clinical changes may relate to changes in cerebral volume and diffusion measures. A negative impact may exist of volume increase of the hippocampus on cognitive outcome, and in one study, a negative impact of decrease of MD in this region [22–24]. Additionally, volume increase of the hippocampus and amygdala has been associated with reduction of depressive symptoms tested over scans between 24 h after the second ECT-session, though combined with scans after the ECT-course [12]. Whether the same changes contribute to both cognitive side-effects and efficacy is unclear. Cerebral volume and diffusion changes assessed in multiple regions directly after initial ECT-sessions may be more specific to the reported associations of efficacy or cognitive side-effects.

In this prospective longitudinal study, we therefore investigated early and later temporal changes in cerebral volume and diffusivity of MDD patients, both at several timepoints throughout the ECT-course and at three months follow-up. Furthermore, we elected to analyze not only the conventional gray matter regions of interest, namely the hippocampus and amygdala, but also the whole-brain white matter. This approach aims to capture neurobiological changes that are not typically examined in previous investigations. Data were corrected for normal test-retest variability based on a group of healthy controls (HC). We subsequently associated the early cerebral changes with cognitive and efficacy measures.

## Methods and materials

### Study design

Data were included from the participants from two prospective studies in depressed patients treated with ECT between 2017 and 2023 (NCT04028596, NL4289) [25–27]. MRI scans of both studies were acquired on the same scanner with identical sequences. To correct for test-retest effects, healthy control (HC) data were included from age-, sex-, and education level [28]-matched participants who were assessed prospectively as part of one of the studies with cognitive tests and MRI (Supplementary Materials). All patients and HC provided oral and written informed consent before entering the studies.

## Participants and timepoints of measurements

**Fig. 1:**
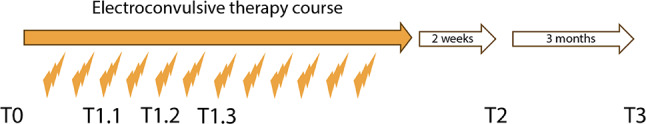
Schematic depiction of the timepoints. T0 = within 2 weeks before start of electroconvulsive therapy (ECT). T1.1 = first magnetic resonance imaging (MRI) directly after median session 3 (interquartile range [IQR] = 2). T1.2 = second MRI directly after median session 5 (IQR = 1.75). T1.3 = third MRI directly after median session 7 (IQR = 2). T2 = MRI within two weeks after completing the ECT-course. T3 = MRI at three months follow-up

ECT-patients were > 17 years-of-age, both hospitalized and non-hospitalized, treatment-resistant, currently depressed and classified (according to the Dutch version of the Mini International Neuropsychiatric Interview (MINI) [29] as unipolar depressive disorder, bipolar disorder or schizoaffective disorder). MRI and clinical data of ECT-patients were gathered before starting of the course at baseline; (T0), postictal ~ 1 h each time after three separate ECT-sessions (for details, see below) (T1; separate as T1.1, T1.2 and T1.3), within two weeks after completing the ECT-course (T2) and at three months follow-up (T3) (Fig. 1).

**Fig. 2:**
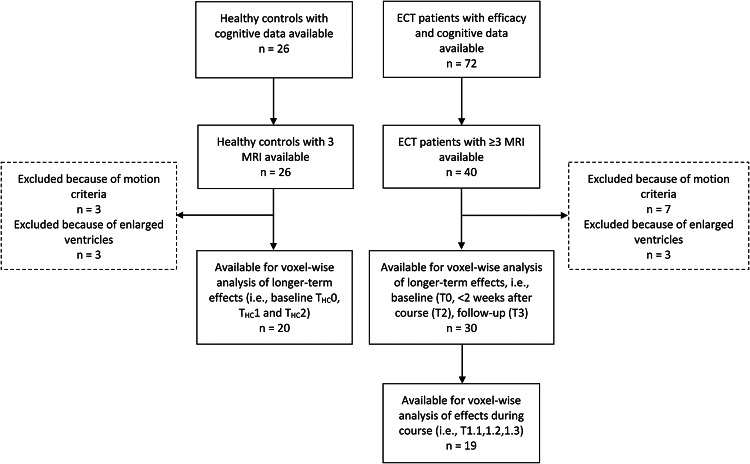
Flowchart of availability of data

The HCs were > 17 years-of-age, all scored negative on all items of the MINI, and were assessed at baseline (T0), after one month (T2) and after three months (T3). 72 ECT-patients could be included. 32 of these patients were excluded due to missing MRI data. Of these,10 did not pass quality control of MRI. A further 11 patients were excluded only for the analysis of MRI during the ECT-course because of missing data (Fig. 2).

### ECT-procedure

ECT-sessions were administered according to Dutch guidelines, two times a week [30], using bifrontotemporal (BL) or unilateral (UL) electrode placement (Supplementary Methods).

### Image acquirement and preprocessing

At identical timepoints as the cognitive tests, T1W and DTI MRI scans were acquired in ECT-patients as well as in HCs (T0, T2 and T3). Additionally, only in the ECT-patients, at a first timepoint during the ECT-course (T1.1; median after 3rd ECT-session [IQR 1.25]), at a second timepoint (T1.2; median after 5th ECT-session [IQR 0.75]) and at a third timepoint (T1.3; median after the 7th ECT-session [IQR 1]), MRI scans were acquired approximately one hour after the ECT-session. Acquisition parameters and preprocessing are described in the Supplementary Methods.

### DTI analyses

We fitted a diffusion tensor model at each voxel (Supplementary Methods). For voxel-wise analysis of WM, we used the Tract-Based Spatial Statistics pipeline (TBSS; [31]), part of FSL [32]. To investigate MD changes in expected areas of GM volume increase [12], we chose ROIs of the hippocampus and amygdala, limited to initial volume change at T1.1. Additionally, we chose the insular cortex because of its relevance for depression [33] and possible volume increases beneath the ECT-electrodes.

### Clinical outcomes

In MDD patients, depression severity was assessed at T0, T2 and T3 with the Hamilton Depression Rating Scale (HDRS-17) [34], while extensive cognitive measurements were performed as well. Global cognitive functioning was assessed using the Montreal Cognitive Assessment (MOCA) [35] or Mini-Mental State Examination (MMSE) [36]. MMSE scores were converted to MOCA using a validated method [37]. More specific cognitive domains were examined with the Rey Auditory Verbal Learning Test (RAVLT; immediate and delayed recall) [38], the Category Fluency of animals and occupations (CFa and CFo; executive retrograde memory) [39], the Letter Fluency (LF; executive retrograde memory) [39], the backwards digit span testing (DSB; working memory) [40], the STROOP task (processing speed) [41], the Trail Making Test A and B (TMT A/B; attention and cognitive flexibility, respectively) [42], and the Subjective Assessment of Memory Impairment (SAMI) [43]. RAVLT, DSB, STROOP, TMT A/B, and SAMI were only available in one of the two included studies.

We first explored changes in cognitive functions over time in the ECT-patients corrected for test-retest variability over time based on the HCs. Thereafter, we selected the tests that significantly changed in patients and used these outcomes to examine associations with (early and later) cerebral volume and diffusion changes.

## Statistical analyses

To investigate changes *during* the ECT-course, we used three planned Helmert contrasts between baseline (T0) and the three available postictal timepoints (i.e., T1.1, T1.2 and T1.3), as well as between the postictal timepoints themselves. To test changes *after* the ECT-course over T0, T2 and T3 HC, we applied polynomial contrasts to assess linear and quadratic trends, and conducted interaction analyses to examine whether these trajectories differed from those of the HC group. Voxel-wise family wise error (FWE) correction was performed using Threshold-Free Cluster Enhancement (TFCE) at a significance level of 0.05. For all analyses, we applied Bonferroni correction for multiple comparisons (i.e., for the cognitive test battery, FWE correction for 11 models). Additionally, effect sizes for the interaction terms were calculated using Pearson’s *r*. Further details are described in the Supplementary Materials.

## Results

### Included sample and clinical characteristics

In total, 72 patients were included in the datasets. We included 30 patients for the analysis of brain changes at T0, T2 and T3, and 19 patients for T0 and T1 (Fig. 2). Table 1 summarizes the characteristics of the included patients and HC. Due to missing data, the number of ECT-patients varied for the individual cognitive tests (see further). At group level, ECT-patients did not differ from HC regarding age, sex and education level (*p* values > 0.05). Patients excluded due to missing or insufficient quality of MRI scans were significantly older (*p* = 0.001; Supplementary Table 1). Over time from T0 to T2 and T3, the mean HDRS-scores showed a significant quadratic decrease ([*n* = 70], *β* = −1.8, *r* = 0.43, *p* < 0.001; Supplementary Fig. 1).

**Table 1:** Population characteristics

	Test for cognitive outcome			Test for changes in MRI data during the ECT course (T1.1, 1.2, 1.3)	Test for changes in MRI data at T2 and T3			
	ECT patients	Healthy controls		ECT patients	ECT patients	Healthy controls		
Variables	With available outcome data n = 72	With available outcome data n = 26	Significance	With available MRI data during ECT course n = 19	With available MRI data after ECT course n = 30	With available MRI data after ECT course n = 20	Significance	Used test
Demographic variables								
Age in years, mean (SD)	52.3 (15.3)	53.0 (15.3)	.84	48.5 (15.3)	45.7 (13.3)	53.0 (14.4)	.08	Unpaired T test
Female sex, n (%)	42 (58.3)	15 (57.7)	.95	13 (68.4)	19 (63.3)	11 (55.0)	.55	Chi-squared test
Education level, median (IQR)	5 (1.0)	6 (1.75)	.13	5 (1)	5 (1.0)	6 (1.25)	.20	Fisher's exact
Diagnosis of MDD, n (%)	58 (80.6)	NA		14 (73.7)	27 (90)	NA		
Diagnosis of BD, n (%)	12 (16.7)	NA		4 (21.1)	3 (10)	NA		
Diagnosis of SA, n (%)	2 (2.8)	NA		1 (5.3)	0 (0)	NA		
Psychotic subtype, n (%)	15 (20.8)	NA		4 (21.1)	5 (16.7)	NA		
ECT variables								
Total number of ECT-sessions during course, median (IQR)	16 (14)	NA		13.5 (8)	17 (13.3)	NA		
Right unilateral electrode position at end ECT-course, n, (%)	17 (23.6)	NA		6 (31.6)	7 (23.3)	NA		
Remission of depression after ECT, n (%)	24 (34.3)	NA		8 (44.4)	10 (33.3)	NA		
Change in HDRS score, mean (SD)	.-10.1 (8.8)	NA		.-9.6 (6.9)	.-9.8 (8.2)	NA		

### Structural volume changes

**Fig. 3:**
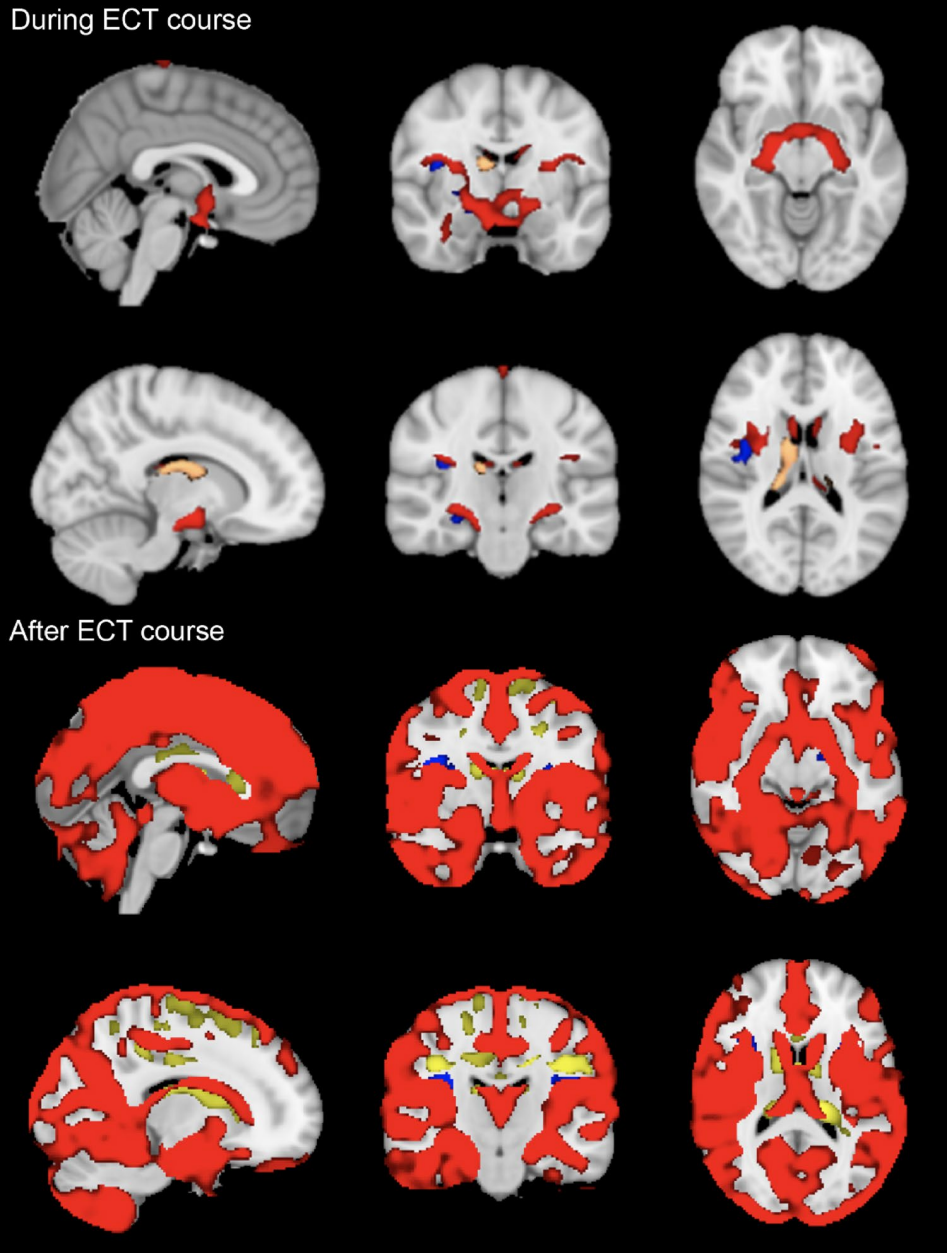
Volume changes in grey (GM) and white matter (WM) segmentation. During the ECT-course, volume increases seem more confined to subcortical GM structures (i.e., hippocampus, amygdala). After the ECT-course, volume increases are generalized over the cortex, though transient after three months (displayed results are a quadratic contrast over T0, T1, and T2). GM increases are depicted in red, WM increases in yellow, and WM decreases in blue. No statistically significant GM decreases were found. All *p two-sided* < 0.05

*During the ECT-course.* The GM of multiple subcortical structures (i.e., hippocampus, amygdala and insular cortices in both hemispheres) increased significantly over the three timepoints during the ECT-course (i.e., average T1.1-T1.3, compared to T0 and corrected for changes over time in HCs; see Fig. 3, Supplementary Video 1). Notably, no significant changes in the cortex were detected. In the WM segmentation, we found significant decreases in volume adjacent to some of the GM increases. The right thalamus, erroneously automatically segmented into WM, significantly increased in volume (Fig. 3). Comparisons between the consecutive timepoints during the ECT-course further showed non-significant (*p* > 0.05) GM increases between T.1.1 and T.1.3.

*After the ECT-course.* Across T0, T2 and T3, we found widespread GM increases of cortical and subcortical structures (Fig. 3, Supplementary Video 2), controlled for HC. These increases were quadratic, and transient (i.e., T3 returning to T0, confirmed with post-hoc t-tests of T0 versus T3, Supplementary Fig. 2) Additionally, some areas in the WM segmentation transiently increased and smaller areas of adjacent WM transiently decreased (Fig. 3).

### Brain diffusion changes

**Fig. 4:**
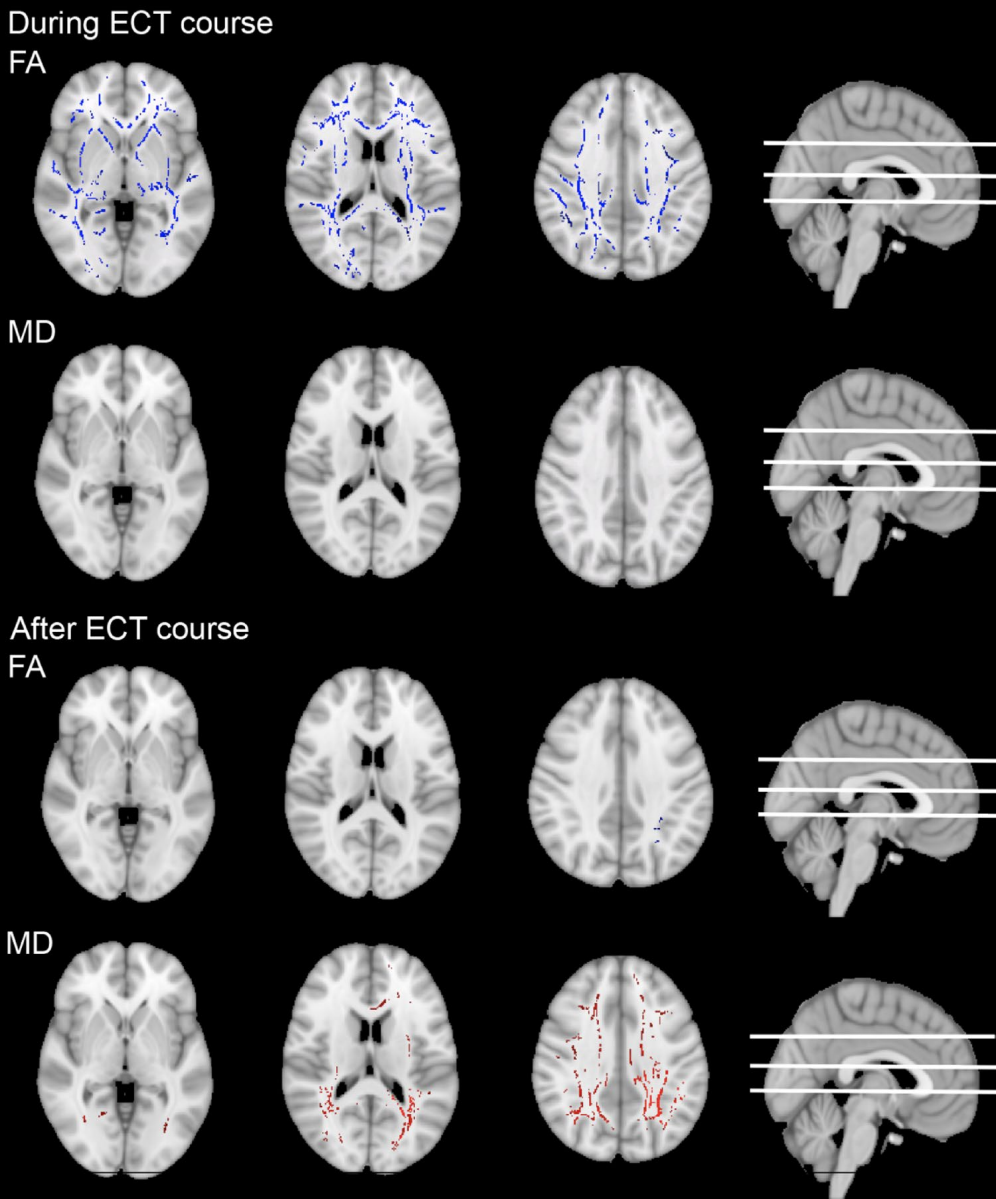
During the ECT course, fractional anisotropy (FA) decreases (blue) in white matter (WM) tracts. In contrast, after the ECT course, FA is largely unchanged, and mean diffusivity (MD) increases (red) linearly over timepoints T2 (< 2 weeks after the ECT course) and T3 (3 months after ECT course). All *p two-sided* < 0.05

*During the ECT-course.* Compared to baseline (T0), we detected lateralized decreases of MD in the right amygdala (*β* = −0.08, *r* = 0.50, *p*_*bonferroni*_ < 0.001) and right hippocampus (*β* = −0.07, *r* = 0.48, *p*_*bonferroni*_ < 0.001) during the ECT-course (i.e., at timepoints T1.1, T1.2 and T1.3), while the contralateral and insular structures did not change (see Supplementary Results). In the WM, MD did not significantly change postictally (at minimum a cluster with *p-*value of 0.051), nor did it change further in the course in the comparisons between postictal measurements. FA though, generalized throughout the WM, was significantly lower postictally (Fig. 4, Supplementary Video 3) [44]. From postictal measurement 1 to 2, FA further decreased in some clusters. From postictal measurement 2 to 3, FA did not further decrease, and instead increased in an occipitoparietal cluster.

**Fig. 5:**
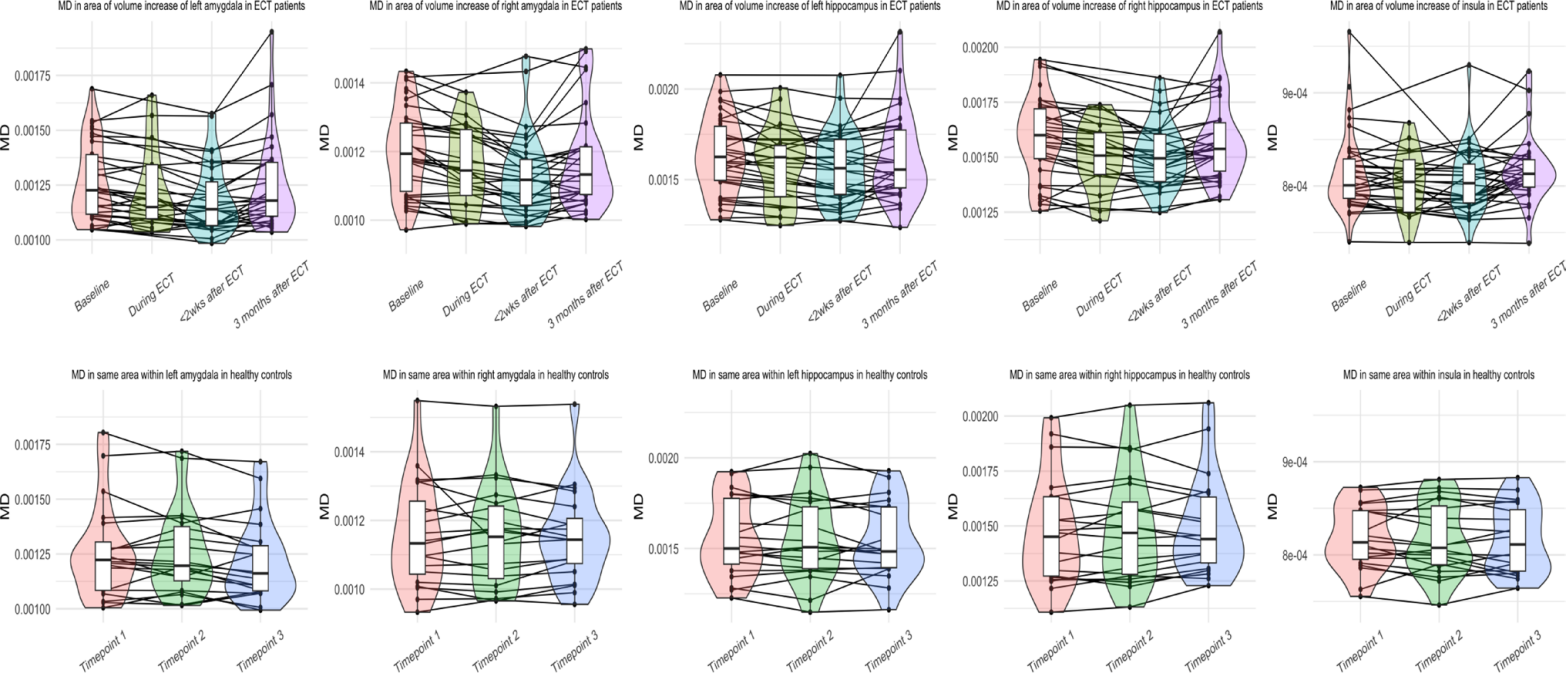
Mean diffusivity significantly decreases in the left and right amygdala, and right hippocampus in ECT patients, but not in the left hippocampus, or insula. Timepoint of during ECT is first MRI sessions during course, i.e. median after 3rd ECT session

*After the ECT-course.* Controlled for changes in HC over time, MD in the left amygdala (*β* = −0.14, *r* = 0.37, *p*_*bonferroni*_ = 0.001), the right amygdala (*β* = −0.12, *r* = 0.29, *p*_*bonferroni*_ = 0.02), and the right hippocampus (*β* = −0.12, *r* = 0.32, *p*_*bonferroni*_ = 0.008) showed transient (quadratic) decreases from baseline (T0) across T2 to T3 (Fig. 5, confirmed with post-hoc t-tests T0 with T3 *p* > 0.05, Supplementary Video 4). Changes in left hippocampus and both insular cortices did not reach statistical significance. In contrast, in the WM, we found persisting, widespread linear increases in MD, while FA was mostly unchanged except for one small cluster showing a linear decrease in FA in the left parietal WM tracts (Fig. 4, Supplementary Fig. 2).

To further investigate the nature of the diffusion changes in WM, we post-hoc applied NODDI-DTI on our single-shell DTI maps [45] to calculate neurite density indices (NDI) and orientation dispersion indices (ODI) from the normalized, non-skeletonized DTI maps. We used the FA distance maps from the TBSS pipeline to project NODDI maps onto the skeleton and found that the MD increases in WM over T0, T1 and T2 were accompanied by linear decreases in NDI, while ODI remained unchanged.

### Neuropsychological outcomes

**Fig. 6:**
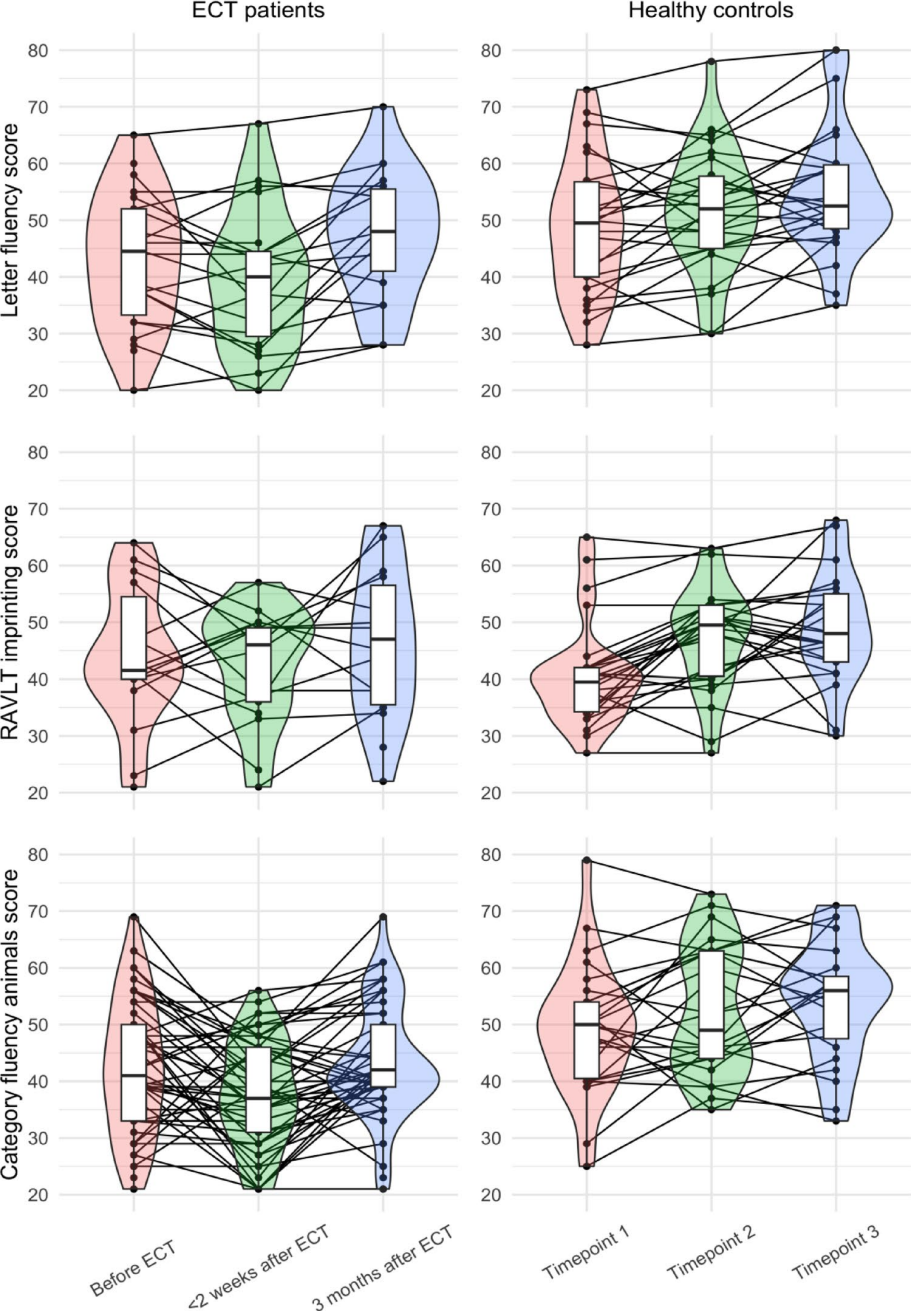
The letter fluency test (*p*_*bonferroni*_ = 0.04), the imprinting sub-score of Rey Auditory Verbal Learning Test (RAVLT; *p*_*bonferroni*_ = 0.04) and the Category Fluency of animals test (*p*_*bonferroni*_ = 0.02) transiently decreased after electroconvulsive therapy, corrected for test-retest in healthy controls

At baseline, ECT-patients scored significantly lower than HC regarding the MOCA (ECT [*n =* 70] = 23.3 ± 5.1; HC = 26.6 ± 2.0, *p* < 0.01), CFa (ECT [*n =* 62] = 42.4 ± 12.0; HC = 49.2 ± 11.4, *p* = 0.02), CFo (ECT [*n =* 62] = 43.5 ± 11.1; HC = 54.1 ± 12.1, *p* < 0.01), STROOP (ECT [*n =* 20] = 46.0 ± 11.7; HC = 53.2 ± 9.8, *p* = 0.03), DSB (ECT [*n =* 20] = 42.3 ± 10.3; HC = 51.9 ± 9.9, *p* < 0.01) and TMT (ECT [*n =* 19] = 42.3 ± 13.3; HC = 50.2 ± 9.5, *p* = 0.03) (see Fig. 6 and Supplementary Fig. 3). LF (*n* ECT = 22) and RAVLT (*n* ECT = 23, both total and imprinting) did not differ at baseline.

Over T0, T1 and T2, corrected for test-retest effects in HCs, we detected transient (quadratic) decreases of the LF test (*β* = −2.0, *r* = 0.30, *p*_*bonferroni*_ = 0.04), the imprinting sub-score of RAVLT (*β* = −2.8, *r* = 0.30, *p*_*bonferroni*_ = 0.04) and the CFa test (*β* = −2.5, *r* = 0.25, *p*_*bonferroni*_ = 0.02). Figure 6 shows these results for ECT-patients and HCs. These cognitive tests had all recovered at the three months follow-up (T3; see Supplementary Results). Scores on the MOCA, SAMI, RAVLT (i.e., recall score corrected for total imprinted words), TMT, STROOP, DSB, and CFo did not change significantly (see Supplementary Fig. 3 and Supplementary Results) at either of the two timepoints after the ECT-course (i.e., T2 and T3, compared to T0 and corrected for test-retest effects in HC).

### Cerebral volume and diffusion changes in relation to cognitive and efficacy outcomes

We included the LF, RAVLT (imprinting) and CFa as cognitive outcome variables to associate with our MRI outcomes, as these were the only ones to significantly change. No significant associations were found between any change in cerebral volume or diffusivity with any of the established deteriorations on these tests (i.e., when testing for associations of change (T0 – T2) of scores in LF, the imprinting sub-score of RAVLT and the CFa test, with both GM and WM volume change (T0 – T1.1), MD change within GM ROIs (T0 – T1.1), and voxel-wise diffusion changes in the WM skeleton (T0 – T1.1).)

Although a lower decrease in the imprinting score of RAVLT seemed to be associated with a larger GM volume increase during the ECT-course (i.e., T0 – T1.1) in multiple clusters, this association just failed to reach significance after accounting for testing multiple brain-behavior associations (minimum *p*_*uncorrected*_ *=* 0.0125, *p corrected for multiple cognitive tests* = 0.05; Supplementary Fig. 4). Also, change in HDRS-scores at T2 compared to baseline T0 was not associated with any of the cerebral volume or DTI parameters.

## Discussion

In this prospective longitudinal study in ECT-patients, our novel findings reveal early microstructural changes during ECT. In GM, we found an acutely decreased diffusivity which is transient within 3 months. In contrast, in WM, we found an increased diffusivity which is longer lasting. Furthermore, we confirm earlier reported macrostructural changes of GM volume increase which is transient within 3 months. Though during the ECT-course, the GM volume increase seems more localized to subcortical structures, which after the ECT-course is generalized over the cortex. The observed micro- and macrostructural changes in patients were notably not significantly associated with the observed transient decline in cognitive functioning, nor with clinical efficacy after ECT.

### Interpretations of microstructural and macrostructural changes in GM

Consistent with earlier findings, we found volume increases of the subcortical GM structures in ECT-treated depressed patients accompanied by decreases in MD [19, 20]. Of these structures, in particular the hippocampus and amygdala are associated with depression [46]. Furthermore, we confirm earlier data showing that these volume increases are already present from the 1 st to 3rd ECT-session [12, 13]. Our novel finding is that the decreases in MD already occur after the third ECT-session.

Multiple interpretations are possible regarding the early reduction in mean diffusivity and increase of gray matter volume. Cellular stress in the form of cytotoxic edema may explain our findings of acutely and relatively short-lived decreased MD, and volume increase, in GM [47, 48]. If present, we hypothesize this neuronal stress to be transient, and much less prominent than the neuropathological and/or cellular changes found after status epilepticus or chronic epilepsy in both rodent models and human postmortem brain, supported by a lack of volume loss, and lack of clinical deleterious effects. Indeed, in contrast, malformations, granule cell dispersion, mossy fiber sprouting, cornu Ammonis (CA) cell loss, and astrogliosis have been reported in hippocampal and extrahippocampal limbic regions of epileptic patients [49, 50]. Additionally, the electroconvulsive shock (ECS) treatment paradigm in rodents as a model for ECT differs from those paradigms that induce epileptic seizures. Rodent models for epilepsy utilize methods such as more extensive electric or chemical kindling, resulting in prolonged and spontaneous seizures with permanent neuropathological changes. In contrast, ECS induces brief, self-limiting seizures, designed to investigate potential therapeutic effects such as dendritic remodeling, without leading to spontaneous seizures or permanent neuropathological changes [51]. Volume and diffusivity changes after ECT could in principle also relate to subtle inflammatory changes and the activation of (astro)glia [52]. ECS initiated an immediate glial reaction, though this was already decreased four weeks later, which may fit our MD findings in GM [53]. Additionally, no indications could be found for substantial cell loss or glial activation in a recent study on hippocampal tissue from (chronic) ECT-treated, depressed patients [54]. Hypothetically, transient neuronal stress may thus cause the transient cognitive side-effects, and consequently induce expression of early-immediate genes and neuroplasticity, leading to reduction of depressive symptoms in ECT (‘disrupt and rewire’) [9, 55].

Alternatively, primarily ensuing neuroplasticity changes have been proposed to be associated with the decreased MD in GM, among others in the hippocampal dentate gyrus [20, 54]. Studies inducing ECS in rodent models reported widespread neuroplasticity changes, e.g. in hippocampal neurogenesis, cortical proliferation and synaptic changes [56, 57], while dendritic arborization and widespread glial activation have been observed after three ECS sessions [58]. The cellular changes in animal models compare to our findings of an early decrease of MD coinciding with volume increase, as neuroplastic effects in GM are associated with decreased MD in the literature [20]. ECT may thus rapidly and transiently cause neuroplastic effects, that appear to be initially concentrated in depression-related subcortical structures.

### Microstructural changes in WM are longer lasting

In contrast to our findings in GM, diffusivity in WM increases and is present longer than 3 months. Because of the differences in diffusivity and longevity of our findings, we hypothesize that we captured multiple processes in the brain throughout the ECT-course and beyond. In WM, we interpret our findings as increased extracellular free diffusion, without significant changes to microstructural organization, because of the absence of significant changes of FA after the ECT-course. Also, though preliminary, this seems supported by our findings of a coinciding decrease of neurite density (NDI), without a change in the orientation variation of neurites (ODI). In a different publication [44] on the same study sample, we find decreased FA shortly after the ECT session, which thus seems very short lived. Whether the longer present increase of diffusivity in WM, hypothetically because of a subtle inflammation, i.e. glial activation and/or increased blood-brain barrier permeability, and consequently adaptive ‘remodeling’ contributes to the efficacy of ECT, has to be further examined in future research.

### Lateralized decreases of MD in GM throughout ECT, but not beyond

We further detected right-sided decreases of MD in GM in the hippocampus and amygdala during the ECT-course, a lateralization that was no more present within two weeks after the ECT-course. Though this may be caused by differences in sample size over timepoints, which compromised our power to detect changes, we hypothesize that the initial lateralization could be due to the right unilateral electrode placement in a quarter of our included patients. Initially, a direct influence of the e-fields on the right side of the brain may cause microstructural changes [59], while after the ECT-course, we speculate this may lead to a cascade of neurotrophic effects, which spreads throughout the brain, again dissipating the lateralization.

### No associations with cognitive outcome and efficacy

In our depressed patients, mood improvement and clinically relevant cognitive side-effects were seen, which were statistically significant at group level. We replicated that the cognitive side-effects are transient after 3 months [60]. We did not detect associations between the transient disturbances in verbal recall and retrograde executive memory functions, and any of the observed MRI changes, nor between antidepressive efficacy and MRI measures. We hypothesize that this results from a lack of power, since voxel-wise regression analyses with a more stringent correction of multiple comparisons, require far larger sample sizes, especially of linear outcome data [61]. We did detect borderline significant associations between the imprinting score of RAVLT and anatomical clusters adhering to the functional default mode and salience networks (Supplementary Fig. 4), suggesting that larger volume increases in these regions mitigate the ECT-related cognitive decline.

### Detected cognitive side-effects of ECT are transient

The current study showed that depressed patients had lower cognitive functioning before ECT compared to healthy controls, probably as part of the disorder. Despite the stigma surrounding cognitive side-effects of ECT, we not only show that the cognitive decreases are transient after three months, we also showed that some cognitive performances improved after ECT. Moreover, we did not detect subjective cognitive side-effects after the ECT-course. Therefore, more research-substantiated information regarding (baseline) cognitive functioning and the to be expected cognitive side-effects should be given to patients, caregivers and involved professionals. This may help decrease ECT stigma.

### Strengths and limitations

Our design has multiple strengths. First, we collected longitudinal data in ECT-patients and matched HC over three months, at several timepoints, as well as during the ECT-course in the patients. We combined multiple modalities of structural MRI data, and scanned all patients and HC in the same environment and MRI scanner. Second, we were able to additionally collect an extensive battery of cognitive functions in ECT-patients as well as in the HCs. However, our design also has limitations. First, our final sample size was relatively small. MRI data was not available for all ECT-patients during the ECT-course (i.e., at T1), which also caused heterogeneity in the number of patients per timepoint. Also, the number of patients per cognitive outcome measure was heterogeneous due to missing data, as well as differing outcome measures between the two included studies. Patients excluded due to missing or insufficient quality of MRI scans were significantly older, possibly because more frail patients were lost to follow-up due to the extra burden of the T3 investigations. Second, because of our relatively hypothesis-free approach, we included a multitude of outcome measures, for which we had to perform stringent FWE correction, ultimately limiting our power to detect associations. Third, apart from MD of amygdala, hippocampus and insula, we employed whole-brain voxel-wise regression to test for associations with cognitive outcome, which is less sensitive than limiting analysis to specific structures. Fourth, we post-hoc aimed to further specify the MD changes in WM using NODDI in our single-shell data, though no consensus exists whether this is feasible without multi-shell acquisitions [45]. Also, while one possible hypothesized mechanism in ECT is inflammatory changes, the NODDI parameters produce mixed results in pathologically confirmed neuronal tissue where glia cells are activated [62].

We therefore advise future studies to use models such as the recently developed soma and neurite density imaging (SANDI) [63], which we were unable to apply, since our data lacked the required multi-shell acquisition up to *b* = 6000s/mm^2^ [64].

In conclusion, ECT in the current cohort improved depressive symptoms until 3 months after cessation, and induced some cognitive side-effects which were transient. Further throughout and beyond the ECT-course, early - but transient – significant brain volume increases were found in subcortical gray matter structures, which were accompanied by – also transient - decreases in (local) mean diffusivity. In contrast, diffusivity increases in white matter tracts seemed to persist for at least three months after ECT. Significant verbal memory disturbances did not associate with early structural or diffusion brain changes. Our findings show that while macrostructural changes are transient, microstructural changes are divergent and longer lasting.

## Supplementary Information

Below is the link to the electronic supplementary material.

Supplementary Material 1

Supplementary Material 2

Supplementary Material 3

Supplementary Material 4

Supplementary Material 5

Supplementary Material 6
