# Supplementary material for: Electroconvulsive therapy induces rapid microstructural and macrostructural changes, but microstructural changes are longer-lasting
Source: Eur Arch Psychiatry Clin Neurosci. 2026 Mar 2;276(4):1355–67. doi: 10.1007/s00406-026-02195-0 (PMC13234036; doi:10.1007/s00406-026-02195-0)
Supplement: Supplementary file 5 — Supplementary Material 5 [file 406_2026_2195_MOESM5_ESM.docx]

Supplementary Methods

*Study design*

Data were included from participants in two prospective studies in patients (approved by the local medical-ethical authority, i.e., METC Arnhem/Nijmegen under no. 2019-5100 and no. 2016-2324, respectively) and one in healthy subjects (ethical approved ERB study number 2020-BC-12375); i.e., participants in the ‘StudY of effect of Nimodipine and Acetaminophen on Postictal Symptoms after ECT’ (SYNAPSE; NCT04028596, a prospective monocenter randomized controlled trial which was described in detail elsewhere (1, 2)), the ‘Study on Predictors of Outcome in ECT Patients’ (SNOEP; NL-OMON43040, a prospective monocenter observational study to predict efficacy and cognitive side-effects using MRI), and the ‘Schema-ECT healthy control project’, in which age-, sex-, and education level (3)-matched participants were assessed prospectively with cognitive tests and MRI to correct for test-retest effects at three separate timepoints. The studies were performed between 2017 and 2023. In all three groups, the same MRI scanner and software in Rijnstate, as well as cognitive battery assessors, were used. All patients and HC provided oral and written informed consent before entering the studies.

*ECT-procedure*

ECT-sessions were administered according to Dutch guidelines, two times a week (4), using the Thymatron IV device (Somatics Incorporation Lake Bluff, Illinois, USA). Electrode placement was bifrontotemporal (BL) or unilateral (UL; regularly right unilateral [RUL] and – exceptionally - left unilateral [LUL]). Treatment was performed with brief-pulse (1.0 ms) and constant-current (900 mA) stimuli, dose-titration-based (i.e., in UL 6 times and in BL 2.5 times seizure threshold) or age-based. During the ECT procedure, patients were pre-oxygenated at 100% oxygen and actively ventilated until resumption of spontaneous breathing. Etomidate (0.2 – 0.3 mg/kg body mass) was routinely used as the sedative agent and succinylcholine (0.5 – 1.0 mg/kg body mass) as muscle relaxant. ECT-courses were terminated when remission was reached, or plateau in mood was shown after two consecutive ECT-sessions, or when no effect was seen after ten BL sessions.

*Image acquirement and preprocessing*

DTI and T1W MRI scans were acquired on a 3T Philips Ingenia MR scanner with a 15 channel Philips dStream HeadSpine coil. First, a T1W brain scan was acquired using a 3D turbo field echo multi-shot with the following parameters: repetition time (TR) = 7.5 ms, echo time (TE) = 4.6 ms, flip angle = 8⁰, field of view (FOV) = 256 x 238 mm, data matrix size = 128 x 128, resolution = 1.1 mm isotropic, slice thickness = 1.77 mm, slice gap = 0 mm, slices = 145. Additionally, single-shot echo-planar imaging was used for the DTI acquisition with the following parameters: TR = 4.58 s, TE = 94 ms, flip angle = 90⁰, FOV = 224 x 224 mm, data matrix size = 512 x 512, resolution = 2 mm isotropic, slice thickness = 2 mm, slice gap = 0 mm. Thirty-two diffusion directions were obtained with a b-value of 1000 s/mm2 and one additional b0 image (in anterior-posterior direction). For the DTI scan, the anterior-posterior commissure line was used to align the FOV. Preprocessing of T1W MRI was performed using the Computational Anatomy Toolbox (5) in Matlab R2022a (6). T1W images were skull-stripped, segmented into cerebrospinal fluid (CSF), GM and WM, and normalized to the Montreal Neurological Institute (MNI) template using Diffeomorphic Anatomical Registration using Exponentiated Lie (DARTEL) algebra (7). Segmented T1W maps were smoothed with an 8 mm^3^ full-width at half-maximum kernel. For DTI, denoising and Gibbs ringing correction were performed (MRtrix version 3.0; (8)). We did not acquire reverse phase-encoded scans and therefore synb0-DisCo was used (Synthesized b0 for diffusion distortion correction (9, 10)). Using this pipeline, an ‘undistorted’ b0 image was synthesized using the b0 image and T1W image to enable susceptibility distortion correction, which was then performed using FSL topup (fMRI brain Software Library (FSL); v6.0.5 (11)). All diffusion-weighted images were corrected for motion and eddy-current distortions using eddy of FSL with b0 image as reference for alignment (“EDDY”; FSL; v6.0.5 (12)). The results of preprocessing were visually checked for erroneous results and quality. Exclusion due to motion artifacts was performed based on visual inspection. Scanner sequences did not differ between studies. MRI scans at T0, T2 and T3 were performed at any time of the day.

*DTI analyses*

To compute FA and MD, a diffusion tensor model was fitted at each voxel using FSL DTIFIT (FSL; v6.0.5). Using the Tract-Based Spatial Statistics pipeline (TBSS; (13)), part of FSL (14), we nonlinearly transformed DTI scans to the FMRIB58 FA template. For the voxel-wise analyses of FA and MD, a mean WM skeleton thresholded at 0.2 was projected onto the DTI maps. Patients with enlarged ventricles were excluded from the voxel-wise analyses, because of CSF overlapping with the skeleton (see Figure 1).

To investigate MD changes in expected areas of GM volume increase, we chose ROIs of the hippocampus and amygdala because of their probable role in cognitive side-effects or efficacy in ECT (15). Additionally, we chose to construct a ROI of the insular cortex because of expected effects of volume increase beneath the ECT-electrodes. Canonical masks were extracted from the Harvard-Oxford Subcortical and Cortical Structural Atlases using a threshold of probability of 0.2 (16). These masks were then limited to areas of T1W volume at T1.1. Normalized, non-skeletonized DTI maps from the TBSS pipeline were overlaid with the masks of our ROIs to compute mean MD values.

*Statistical analyses*

Descriptive statistics of the included variables were performed and presented as frequencies and percentages for categorical variables, means and standard deviations (SD) for normally distributes continuous variables, and medians and interquartile range (IQR) for non-normally distributes variables. Chi squared test was performed to test differences in sex between groups.

*During the ECT-course.* We investigated changes in T1W segmented MRI and MD within ROIs measures by using three planned Helmert contrasts between baseline (T0) and the three available postictal timepoints (i.e., T1.1, T1.2 and T1.3), as well as between the postictal timepoints themselves.

*After the ECT-course.* For the timepoints of T0, T2 and T3, we used polynomial contrasts to test whether there were linear or quadratic changes in T1W segmented, WM skeletonized DTI data, or change of MD within ROIs in the ECT-patients corrected for HC.

*ECT outcome measures.* To test for baseline differences between ECT-patients and HC, we used unpaired t-tests. To test whether the HDRS-scores changed significantly over the timepoints T0, T2 and T3, we used linear mixed-effects models. To test for differences in cognitive change in ECT-patients over time T0, T2 and T3, corrected for test-retest variability in HC between T0, T2 and T3, we used mixed-effects models including all cognitive outcome measures, with patient as random effect, and time by group interactions with polynomial contrasts to test for a linear or quadratic relationship.

*Associations of early changes during the ECT course with cognition.* We first selected relevant cognitive measures that changed over time in the ECT patients compared to HC to limit multiple comparisons problems. The MOCA was included because of its current role as screener for global cognitive functioning in daily ECT-practice. Correlations between T1W segmented and WM skeletonized DTI data with cognitive measures after the ECT-course (T2) were tested using multiple linear regression analyses. We used difference images, or difference scores in case of MD within ROIs, of the first timepoint during the ECT-course (T1.1) minus T0.

*Corrections and multiple comparisons.* Analyses of change in clinical data and MD within ROIs were performed using R version 4.1.1 (17). In all analyses we applied correction for age and sex. The package *lmertest* was used for mixed-effects models (18). Voxel-wise analyses were conducted in Permutation Analyses of Linear Models (PALM) (19). Family wise error (FWE) correction was performed using Threshold-Free Cluster Enhancement (TFCE) at a significance level of 0.05. For all analyses, we applied Bonferroni correction for multiple comparisons (i.e., for the cognitive test battery, FWE correction for 11 models). Additionally, effect sizes for the interaction terms were calculated using Pearson’s *r*.


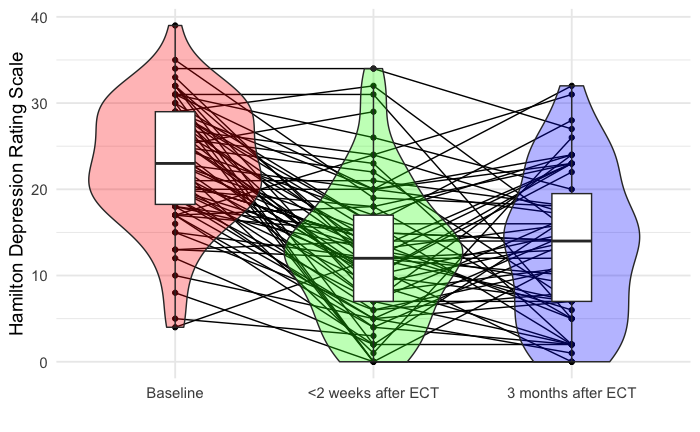


Supplementary figure 1. Decrease of Hamilton Depression Rating Scale score over both timepoints after electroconvulsive therapy (ECT; *p <* 0.01), i.e., after the initial ECT-course and at three months follow-up.


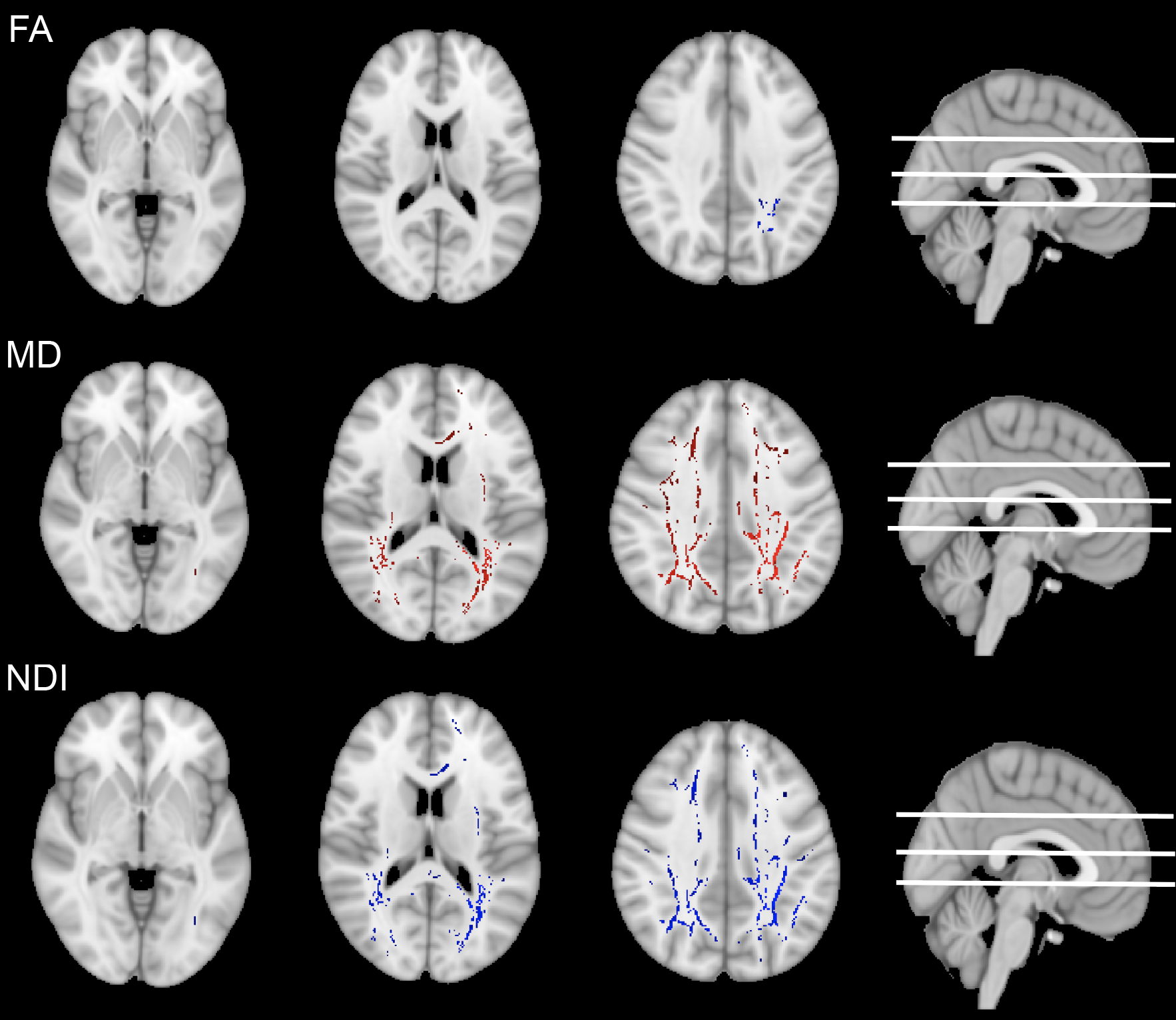


Supplementary Figure 2. Post-hoc t-tests of baseline (T0) versus 3 months after ECT (T3). Orientation dispersion index (ODI), and volume of white matter and gray matter did not significantly change (not displayed). Decrease is displayed as blue, increase as red.

Supplementary Results.

During the ECT-course, compared to baseline (i.e., T1.1, T1.2 and T1.3, compared to T0), we found no decreases of MD in the left amygdala (*β* = -0.02, *r* = 0.17, *p_bonferroni_* = 1.0) and left hippocampus (*β* = -0.02, *r* = 0.13, *p_bonferroni_* = 1.0).

Over T0, T1 and T2, SAMI (*n* = 29) did not significantly change, tested for a quadratic (*β* = 0.15, *r* = 0.16, *p_bonferroni_* = 1.0) and linear contrast (*β* = 0.07, *r* = 0.04, *p_bonferroni_* = 1.0).


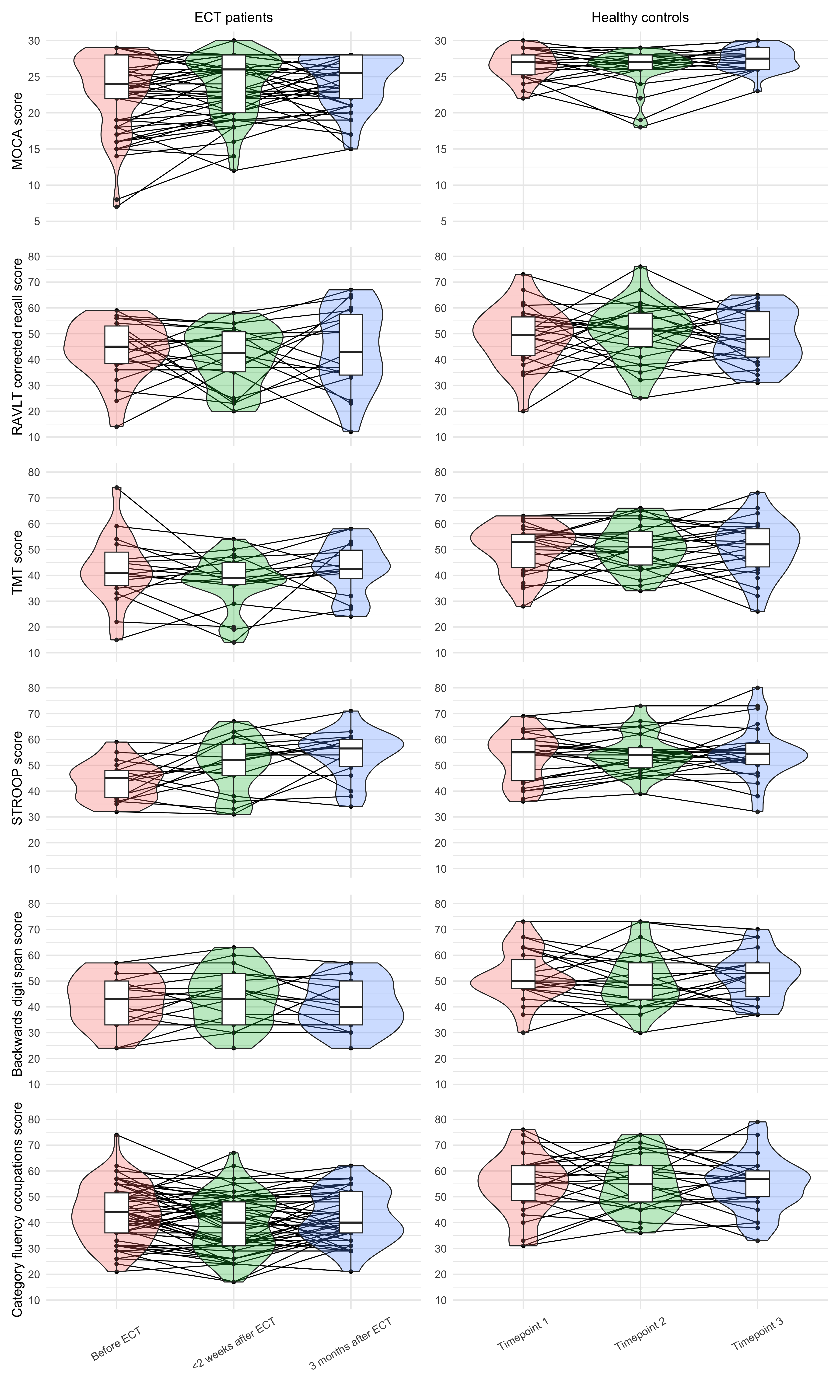


Supplementary Figure 3. Cognitive tests which did not differ in change over time between ECT patients and HC.


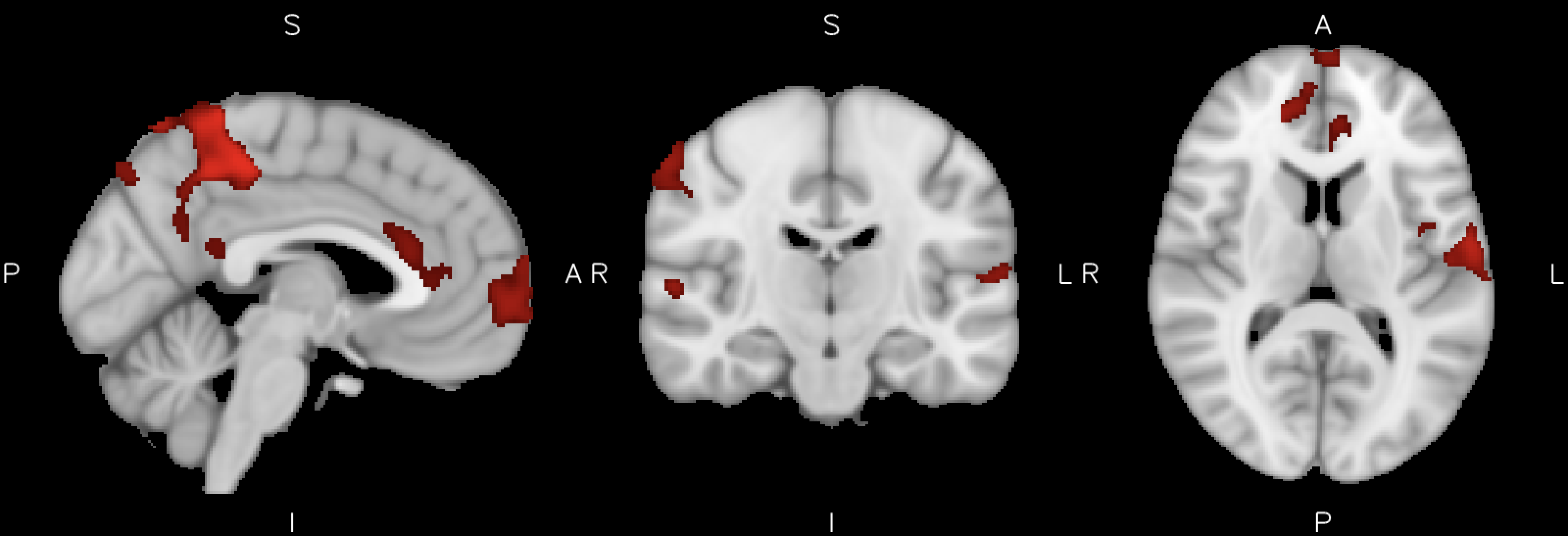


Supplementary Figure 4. Correlation between grey matter increase during the ECT-course and better outcome of imprinting score of Rey’s Auditory Verbal Learning Test. Red areas are *p* < 0.05, family wise error corrected for multiple testing of voxels, but uncorrected for multiple testing of the cognitive outcomes.

References

1. Pottkämper JCM, Verdijk JPAJ, Stuiver S, Aalbregt E, ten Doesschate F, Verwijk E, et al. Acetaminophen or nimodipine to improve postictal recovery after electroconvulsive therapy-induced seizures: a randomized crossover trial. Under review.

2. Verdijk JPAJ, Pottkämper JCM, Verwijk E, van Wingen GA, van Putten M, Hofmeijer J, et al. Study of effect of nimodipine and acetaminophen on postictal symptoms in depressed patients after electroconvulsive therapy (SYNAPSE). Trials. 2022;23(1):324.

3. Verhage F. Intelligentie en leeftijd bij volwassenen en bejaarden: Koninklijke Van Gorcum; 1964.

4. Psychiatrie NVv. Richtlijn Electroconvulsietherapie (ECT): Federatie Medisch Specialisten; 2021 [

5. Gaser C, Dahnke R, Thompson PM, Kurth F, Luders E, Initiative AsDN. CAT – A Computational Anatomy Toolbox for the Analysis of Structural MRI Data. bioRxiv. 2022:2022.06.11.495736.

6. Natick MTMI. MATLAB. R2022a ed2022.

7. Ashburner J. A fast diffeomorphic image registration algorithm. Neuroimage. 2007;38(1):95-113.

8. Tournier JD, Smith R, Raffelt D, Tabbara R, Dhollander T, Pietsch M, et al. MRtrix3: A fast, flexible and open software framework for medical image processing and visualisation. Neuroimage. 2019;202:116137.

9. Schilling KG, Blaber J, Hansen C, Cai L, Rogers B, Anderson AW, et al. Distortion correction of diffusion weighted MRI without reverse phase-encoding scans or field-maps. PLoS One. 2020;15(7):e0236418.

10. Schilling KG, Blaber J, Huo Y, Newton A, Hansen C, Nath V, et al. Synthesized b0 for diffusion distortion correction (Synb0-DisCo). Magn Reson Imaging. 2019;64:62-70.

11. Andersson JL, Skare S, Ashburner J. How to correct susceptibility distortions in spin-echo echo-planar images: application to diffusion tensor imaging. Neuroimage. 2003;20(2):870-88.

12. Andersson JLR, Sotiropoulos SN. An integrated approach to correction for off-resonance effects and subject movement in diffusion MR imaging. Neuroimage. 2016;125:1063-78.

13. Smith SM, Jenkinson M, Johansen-Berg H, Rueckert D, Nichols TE, Mackay CE, et al. Tract-based spatial statistics: voxelwise analysis of multi-subject diffusion data. Neuroimage. 2006;31(4):1487-505.

14. Smith SM, Jenkinson M, Woolrich MW, Beckmann CF, Behrens TE, Johansen-Berg H, et al. Advances in functional and structural MR image analysis and implementation as FSL. Neuroimage. 2004;23 Suppl 1:S208-19.

15. Li J, Cao D, Yu S, Xiao X, Imbach L, Stieglitz L, et al. Functional specialization and interaction in the amygdala-hippocampus circuit during working memory processing. Nature Communications. 2023;14(1):2921.

16. Desikan RS, Ségonne F, Fischl B, Quinn BT, Dickerson BC, Blacker D, et al. An automated labeling system for subdividing the human cerebral cortex on MRI scans into gyral based regions of interest. Neuroimage. 2006;31(3):968-80.

17. Team RC. R: A language and environment for statistical computing. Foundation for Statistical Computing, Vienna, Austria; 2022.

18. Kuznetsova A, Brockhoff PB, Christensen RHB. lmerTest Package: Tests in Linear Mixed Effects Models. Journal of Statistical Software. 2017;82(13):1 - 26.

19. Winkler AM, Ridgway GR, Webster MA, Smith SM, Nichols TE. Permutation inference for the general linear model. NeuroImage. 2014;92:381-97.
